# P-911. Comparison of antimicrobial spectrum coverage score-based antimicrobial consumption in acute care hospitals in an eight-year period: a multicentric retrospective study

**DOI:** 10.1093/ofid/ofaf695.1117

**Published:** 2026-01-11

**Authors:** Shutaro Murakami, Takuhiro Kosugi, Yasuharu Tokuda, Akihito Ito, Tetsuma Hata, Yoshihiro Hamamoto, Hideo Shiohira, Tomoharu Wakugawa, Yasuhiro Sasaki, Hitoshi Honda

**Affiliations:** Tokyo Metropolitan Tama Medical Center, Fuchushi, Tokyo, Japan; Yuuai Mecical Center, Tomigusukucity-Yone, Okinawa, Japan; Muribushi Project for Teaching Hospitals, Urasoe, Okinawa, Japan; Northern Okinawa Medical Center, Nago City, Okinawa, Japan; Tokyo Metropolitan Bokutoh Hospital, Sumida, Tokyo, Japan; Urasoe General Hospital, Urasoe city, Okinawa, Japan; Showa Pharmaceutical University, Machida, Tokyo, Japan; Okinawa Kyodo Hospital, Naha, Okinawa, Japan; Japan institute for Health Security, Hino, Tokyo, Japan; Fujita Health University School of Medicine , Toyoake, Aichi, Japan

## Abstract

**Background:**

Advancing antimicrobial stewardship programs (ASP) has led to various metrics for assessing antimicrobial consumption. Since introducing the antimicrobial spectrum index (ASI) in 2017, similar metrics, such as days of antibiotic spectrum coverage (DASC), have emerged to better evaluate antimicrobial use, particularly considering de-escalation process. This study assessed antimicrobial consumption with a de-escalation process using DASC in 12 hospitals across three regions in Japan.

**Table 1.**
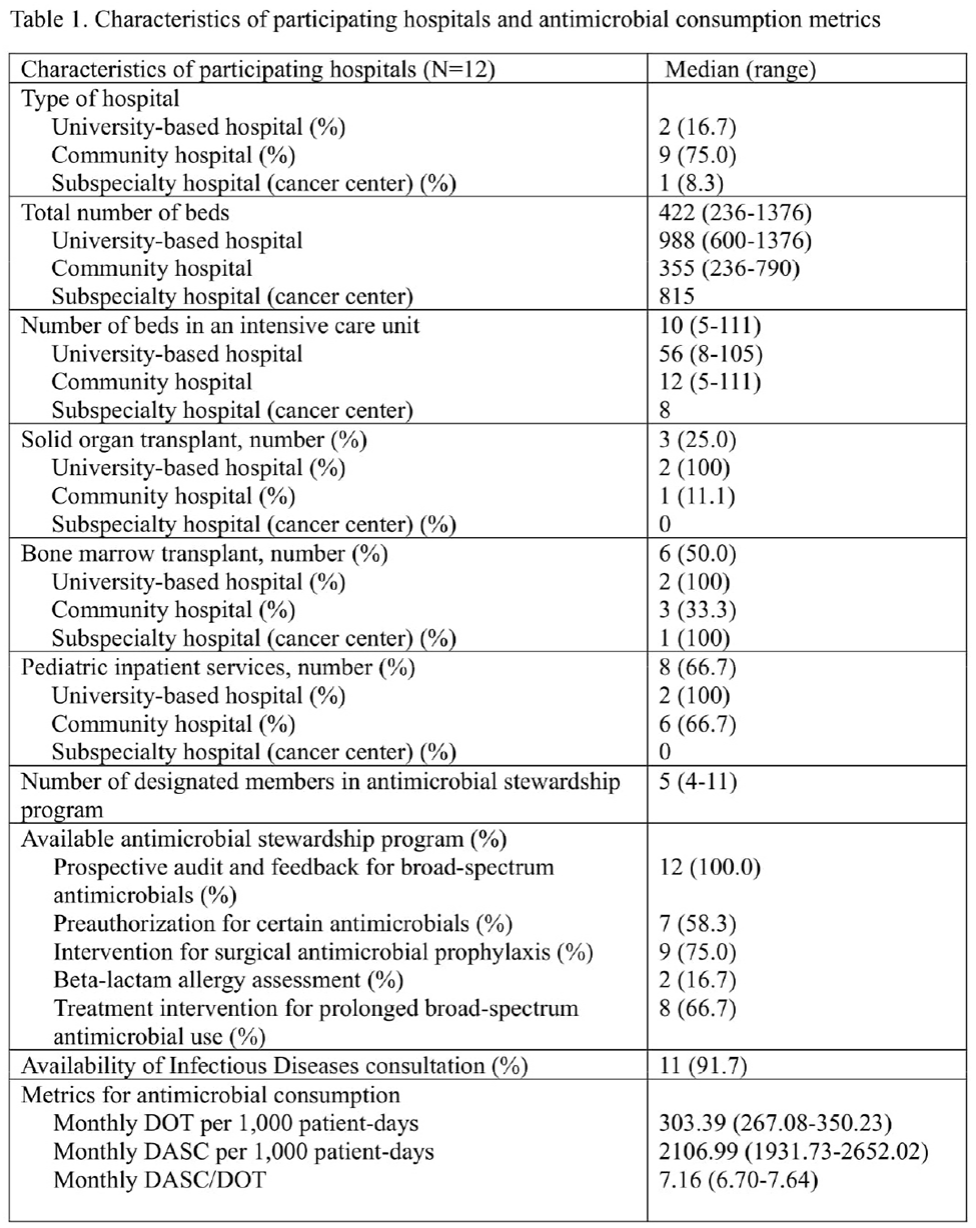
Characteristics of participating hospitals

**Figure 1.**
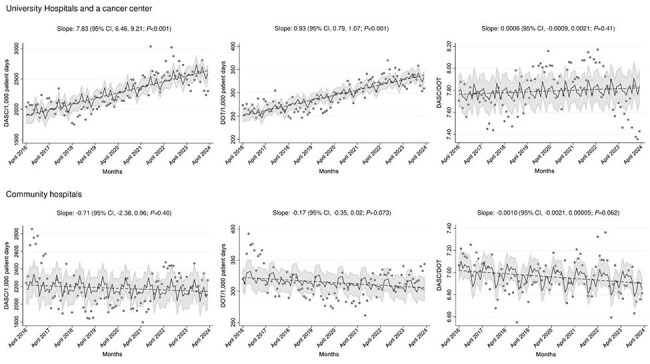
Trend of monthly DASC/DOT and DOT per 1,000 patient-days stratified by hospital types.

**Methods:**

Intravenous antimicrobial consumption data from 12 Japanese hospitals (April 2016–March 2024) were collected. The participating hospitals were located in three regions: West (Okinawa, n=6), East (Tokyo, n=5), and Midwest (Aichi, n=1). Monthly antimicrobial consumption was expressed as days of therapy (DOT) and then converted to DASC per 1,000 patient-days (PD). Hospital-wide de-escalation was evaluated using DASC/DOT. The correlation between DASC/DOT (a new metric) and DOT per 1,000 PD (a conventional metric) was assessed using Spearman’s rank-order correlation test. Hospital characteristics, including ASP availability, and infectious disease consultation, were also collected.

**Figure 2.**
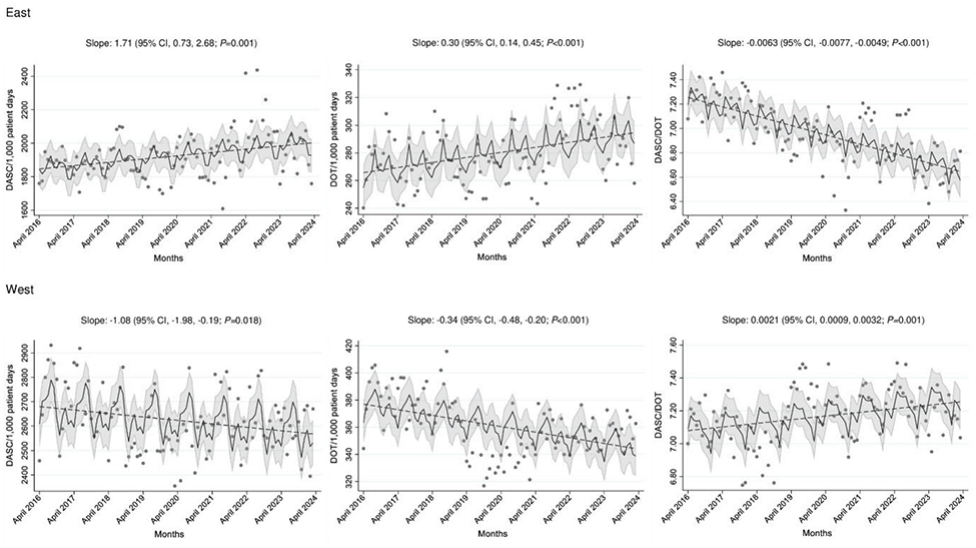
Trends of DOT per 1,000 PD, DASC per 1,000 PD and DASC/DOT stratified by hospital regions

**Results:**

A total of 96 months of data were analyzed. Monthly median DOT, DASC, and DASC/DOT were 303.4, 2107.0, and 7.2, respectively. There was little correlation between DASC/DOT and DOT per 1,000 PD (rho=0.11; *P*< 0.73). University hospitals (median 7.7) and a cancer center (median 8.0) had higher DASC/DOT than the remaining 9 community hospitals (median 6.9), likely owing to the availability of transplant services and a larger number of ICU beds (Figure 1 and Table 1). A substantial decrease in DASC/DOT at one university hospital since August 2022 was observed, likely due to implementing a hospital-wide restriction of carbapenem use. Median DASC/DOTs in East and West were 6.9 and 7.2, respectively, suggesting that the de-escalation process has progressed more at hospitals in Tokyo than at hospitals in Okinawa (Figure 2).

**Conclusion:**

DASC/DOT, showing little correlation with traditional DOT per 1,000 PD, is a useful metric to assess antimicrobial use and de-escalation process. A regional-level and hospital type-level comparisons can help identify facilities needing further ASP promotion.

**Disclosures:**

All Authors: No reported disclosures

